# Exploring ultrafast threshold switching in In_3_SbTe_2_ phase change memory devices

**DOI:** 10.1038/s41598-019-55874-5

**Published:** 2019-12-17

**Authors:** Nishant Saxena, Christoph Persch, Matthias Wuttig, Anbarasu Manivannan

**Affiliations:** 1 Discipline of Electrical Engineering, Indian Institute of Technology Indore, Indore, 453552 Madhya Pradesh India; 2 I. Physikalisches Institut (I.A.) and JARA-Fundamentals of Future Information Technology, RWTH Aachen University, 52056 Aachen, Germany; 3 Department of Electrical Engineering, Indian Institute of Technology Madras, Chennai, 600036 Tamil Nadu India

**Keywords:** Electronic devices, Information storage

## Abstract

Phase change memory (PCM) offers remarkable features such as high-speed and non-volatility for universal memory. Yet, simultaneously achieving better thermal stability and fast switching remains a key challenge. Thus, exploring novel materials with improved characteristics is of utmost importance. We report here, a unique property-portfolio of high thermal stability and picosecond threshold switching characteristics in In_3_SbTe_2_ (IST) PCM devices. Our experimental findings reveal an improved thermal stability of amorphous IST compared to most other phase change materials. Furthermore, voltage dependent threshold switching and current-voltage characteristics corroborate an extremely fast, yet low electric field threshold switching operation within an exceptionally small delay time of *less than 50* picoseconds. The combination of low electric field and high speed switching with improved thermal stability of IST makes the material attractive for next-generation high-speed, non-volatile memory applications.

## Introduction

At present, there is an ever increasing demand for storing huge amounts of data. This is due to advancements in various key areas including embedded systems and the Internet of things (IoT), which enables interactions among various electronic devices used in our daily life. The huge amount of interconnected data demands not only high memory density, but also better programming speed, and therefore a “universal memory” with all-encompassing features such as high speed, high density, low power consumption and non-volatility is highly desirable. In this context, phase change memory (PCM) has already demonstrated tremendous potential. Chalcogenide-based phase change materials offer rapid and reversible phase transitions between high-resistance amorphous (binary 0) and low resistance crystalline (binary 1) states^1–3^. Also, PCM provides high density data storage solutions by means of their ability of multi-bit data storage^4^ owing to stable multiple resistance levels between amorphous and crystalline phases. In addition, it is feasible to vertically stack memory arrays^5^. Further, long term data retention and good endurance can be achieved with materials possessing high thermal stability^6^. Thus, improved device performance can be realized by usage of superior phase change materials. This partly explains the resurgent research interest devoted to explore novel materials with an attractive property portfolio combining good thermal stability, large resistance contrast and ultra-fast switching speed^7,8^.

Conventional phase change materials such as Ge_2_Sb_2_Te_5_ and Ag_5_In_5_Sb_60_Te_30_ offer promising features for memory applications, however, possess relatively poor thermal stability as evidenced by their low crystallization temperatures (150–180 °C)^9^. Besides these conventional materials, In:Sb:Te alloys have also been explored for high thermal stability as well as large resistance contrast for multi-bit data storage^10^. Upon annealing, these alloys show a rapid transition into a single crystalline phase when the composition is close to stoichiometric In_3_SbTe_2_ (IST312) phase, whereas a deviation from the ideal composition may lead to phase separation into InSb and InTe^11^. Therefore, in order to achieve improved device characteristics, it is essential to utilize an In:Sb:Te composition close to the IST312 phase. Furthermore, IST possesses a rock-salt structure similar to materials along the GeTe-Sb_2_Te_3_ tie-line^12^ supporting a rapid crystallization process without requiring major rearrangement of atoms as discussed below. Moreover, IST also offers excellent scaling and multiple resistance states promising for the high-density multi-level data storage^4,13,14^. Thus, owing to its desirable attributes of rapid crystallization, improved thermal stability, scaling and multi-resistance levels, IST shows considerable potential to realize memory devices with universal characteristics, thus promising to enable ultrafast programming capabilities.

In PCMs, programming is accomplished by means of ns short electrical pulses, which induce structural changes between the amorphous (reset) and the crystalline (set) states of phase change material^9,15–17^. The programming speed of PCMs largely depends on the set operation, in which the crystallization process is initiated by threshold switching^18,19^. This switching mechanism is characterized by a rapid breakdown of electrical resistance, which takes place at a critical voltage, known as threshold voltage, *V*_*T*_. Subsequently, Joule heating causes an abrupt increase in device current, *I*_*d*_ leading to the formation of the set state^20^. Threshold switching is known to occur after a finite delay time, *t*_*d*_^18,21^ measured as the time duration between the device experiencing *V*_*T*_ and a sharp rise in *I*_*d*_^16,22^. *t*_*d*_ is an important parameter that depends on the amplitude of the applied voltage pulse, *V*_*A*_. The speed of threshold switching is, therefore, governed by voltage-dependent threshold switching dynamics. Efforts have been made in the past to enhance the speed of threshold switching and set operation in various chalcogenide based PCM devices varying from hundreds of nanoseconds down to the sub-nanosecond timescale^15,17,23–27^. Despite the technological importance of IST for multi-bit data storage, the switching capabilities of stoichiometric IST have not yet been investigated on the picosecond (ps) timescale. Thus, a systematic study of voltage-dependent threshold switching dynamics of IST devices and their delay time characteristics on the ps-timescale are reported here.

In this study, we have employed temperature-dependent resistivity measurements in order to explore material properties such as the thermal stability and resistivity contrast of IST thin films. Subsequently, the threshold switching dynamics of IST devices have been investigated over a wide range of pulse widths ranging from ~10^−3^ s down to ~10^−9^ s, while the voltage-dependent delay time characteristics are obtained on the ps-timescale.

## Results and Discussion

**Figure 1:**
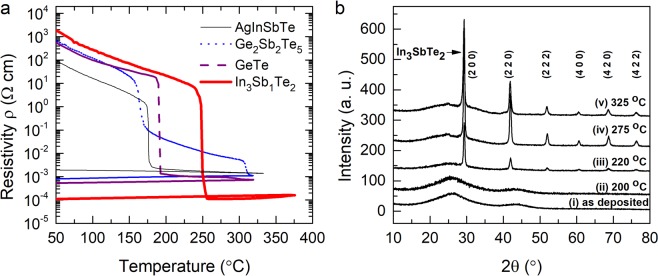
Thermal stability of amorphous state. (**a**) Temperature dependent resistivity of a 74 nm IST thin films (red color solid thick line) compared to 100 nm AgInSbTe (black color solid thin line), 100 nm Ge_2_Sb_2_Te_5_ (blue color dotted line), and 80 nm GeTe thin films (purple color dashed line), revealing a higher crystallization temperature of IST. (**b**) GI-XRD scans of thin IST films as deposited and upon various annealing temperatures up to 325 °C.

The stability of the amorphous state and the resistance contrast between the amorphous and crystalline states of IST are explored by utilizing a temperature-dependent resistivity measurement setup employing the Van-der-Pauw technique^28^. Upon heating the as-deposited thin films, the resistivity decreases smoothly with increasing temperature, reflecting the semiconducting nature of the sample. Upon a further increase in temperature, a drastic drop occurs in their resistivity at around 250 °C, owing to the onset of crystallization. As can be noticed from Fig. 1a, IST thin films are superior to GeTe, Ge_2_Sb_2_Te_5_ and Ag_5_In_5_Sb_60_Te_30_ thin films in several ways. They possess a higher crystallization temperature, and a larger electrical contrast between the amorphous and the crystalline state. Thus, IST possesses an improved thermal stability as evidenced by the higher crystallization temperature (250 °C), which favors long term data retention. Furthermore, a larger electrical resistivity contrast of more than 6 orders of magnitude between the crystalline and the amorphous phase is promising for multi-level data storage applications. Temperature dependent resistivity measurements of IST for various film thicknesses (37 nm to 280 nm) show a pronounced dependence of the phase transition/crystallization temperature upon varying film thickness (see Supplementary Information, Fig. S1). The increase of the crystallization temperature (T_c_) for low thicknesses might be beneficial for scaling. Figure 1b displays grazing incidence x-ray diffraction (GIXRD) patterns of thin films annealed up to 325 °C. In each case, the 74 nm thin IST films are annealed for 15 min with a heating rate of 5 K/min. It can clearly be seen from Fig. 1b that the as deposited films remain amorphous up to an annealing temperature of about 200 °C. Figure 1b reveals that film starts to crystallize into a single IST312 phase at an annealing temperature of 220 °C. It is worth noting that this single IST312 phase structure remains stable up to an annealing temperature of 325 °C (beyond this temperature, disassociation and evaporation of atoms lead to phase separation into InSb and InTe). The high thermal stability in conjunction with single phase crystallization of IST has motivated further systematic studies of the ultrafast switching dynamics of IST devices.

**Figure 2:**
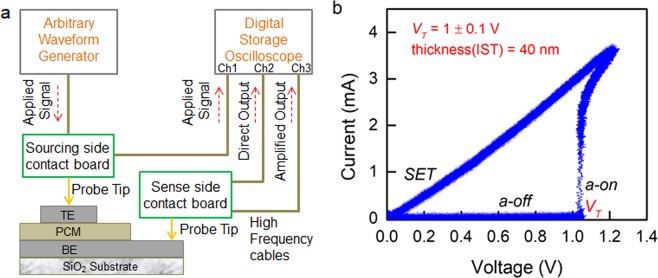
Threshold switching using electrical pulse measurement. (**a**) Schematic of PET setup with sandwich type device architecture. (**b**) Typical Current-Voltage characteristics of IST device indicating threshold switching from a-off to a-on at *V*_*T*_ of 1 ± 0.1 V.

Time-resolved current-voltage characteristics and threshold switching dynamics of IST device have been extensively studied using an advanced Programmable Electrical Test (PET) system, specially designed for investigating ultrafast switching dynamics of memory devices at the ps-timescale^16,29^. A schematic diagram of the experimental setup is shown in Fig. 2a. An arbitrary waveform generator (AWG) is used to generate voltage pulses (with rise time, fall time of 1 ns and pulse-width down to 1.5 ns measured as full-width-half-maximum, FWHM) that are applied to a PCM device. The corresponding real-time response is captured by a digital storage oscilloscope (DSO) with a resolution of 50 ps.

The threshold voltage, *V*_*T*_ is an important parameter that defines the minimum voltage required to switch the device from the high-resistance off-state to the conducting on-state in the amorphous phase. *V*_*T*_ is measured by applying a voltage pulse with longer leading and trailing edges, i.e. triangular shaped pulse. Therefore, we have applied a triangular voltage pulse, having an amplitude of 1.2 V. The resulting current-voltage characteristics of the IST device is shown in Fig. 2b. Initially, the device is in the as-deposited amorphous state where a negligibly small *I*_*d*_ flows through the device until the applied voltage, *V*_*A*_ reaches *V*_*T*_ and thereafter a rapid increase in *I*_*d*_ is seen. This clearly demonstrates threshold switching of the IST device from *‘a-off’* to *‘a-on’* state at *V*_*T*_ of 1 ± 0.1 V (corresponding to a threshold electric field, *E*_*T*_ of 25 ± 2 V/µm, see Supplementary Figure S5). The rapid increase in *I*_*d*_ is due to the abrupt drop in the electrical resistivity of the IST material that subsequently causes Joule heating induced crystallization (see Supplementary Figure S6).

In order to explore the ultrafast switching dynamics of IST devices, careful optimization of voltage pulse parameters is essential. The precise measurement of transient parameters involved in threshold switching such as *t*_*d*_ requires a voltage pulse with a steep leading edge. Therefore, the rise and fall time of the voltage pulse are kept at 1 ns, while the amplitude is systematically varied from 1.1 V to 2.0 V in order to investigate voltage-dependent transient parameters including the delay time characteristics of the IST device. For voltage pulses of *V*_*A*_ close to (but above) *V*_*T*_, a longer pulse-width is required. However, for higher *V*_*A*_, the pulse-width can be reduced. Therefore, we have employed different pulse-widths, i.e. 1 ms for *V*_*A*_ of 1.1 V-1.3 V, 10 µs for 1.5 V, 100 ns for 1.6 V-1.7 V and 5 ns for 1.8 V-2.0 V.

**Figure 3:**
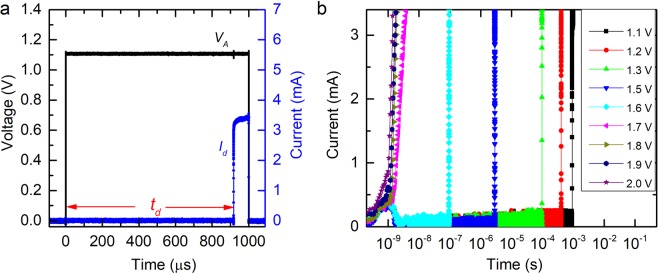
Time-resolved measurement of *I*_*d*_ for a systematic increase in *V*_*A*_. (**a**) Shows threshold switching event with a longer switching delay. (**b**) Systematic increase in applied voltage results in significantly faster switching events.

Figure 3a displays time-resolved current-voltage characteristics of an IST device for a voltage pulse having *V*_*A*_ of 1.1 V (rise time/fall time of 1 ns and pulse-width of 1 ms). It can be clearly observed from Fig. 3a that upon application of *V*_*A*_ of 1.1.V, the device switches within the plateau region of the pulse after a finite *t*_*d*_ of ~910 µs (also, see Supplementary Figure S7). It is remarkable to further note that prior to the threshold switching event, *I*_*d*_ remains fairly constant (also, see Supplementary Figure S8) for the entire duration of *t*_*d*_ corroborating the electronic nature of the threshold switching process. This is, different from a previous report^27^, where a minimal increase in *I*_*d*_ during *t*_*d*_ has been reported, which is possibly related to threshold switching from the melt-quenched amorphous phase in this study.

Thus, the time before threshold switching (*t*_*d*_) can be correlated to the time taken by charge carriers to travel from one electrode to the other forming a conducting path through the amorphous layer in the presence of *E*_*T*_^19^. Upon realizing a critical electric field, the switching event is initiated in the vicinity of one of the electrodes. Since, amorphous chalcogenide semiconductors possess a large number of trap states, under the influence of the electric field, charge carriers fill the trap states leading to enhanced mobility in that region. Further, this decreased resistance region travels through the film thickness to the other electrode and results in a sharp increase in current, i.e. the threshold switching event. Figure 3b demonstrates threshold switching events over a wide range of times upon a systematic increase in *V*_*A*_ from 1.1 V to 2.0 V. It can be observed from Fig. 3b that with an increase in *V*_*A*_, threshold switching speeds up by 6 orders, varying from ~10^−3^ s to ~10^−9^ s. Such a reduction in *t*_*d*_ upon application of higher voltages could be due to the higher mobility of charge carriers in the presence of high electric fields. These charge carriers now travel across the chalcogenide layer filling the trap states and collect at the other electrode much more rapidly leading to a significant reduction in *t*_*d*_.

**Figure 4:**
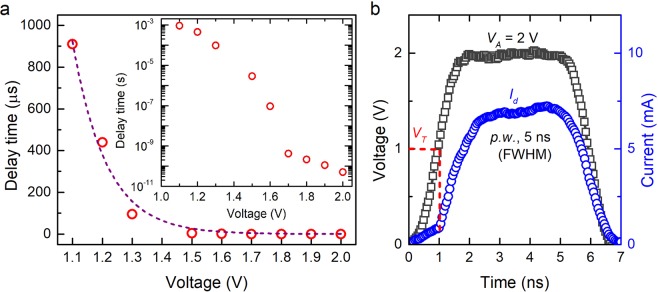
Voltage dependent delay time characteristics. (**a**) Measured delay time reduces exponentially with increase in *V*_*A*_. *t*_*d*_ reduces from 910 µs (for 1.1 V) down to sub-50 ps (for 2.0 V). (**b**) Ultrafast threshold switching at *V*_*T*_ (1.0 V) with *t*_*d*_ < 50 ps for applied voltage of 2 V.

*t*_*d*_ is an important parameter that governs the programming speed of PCMs. At the ps-time scale, it requires a precise measurement while considering the pulse-parameters of the applied voltage pulse. For each measurement of *V*_*A*_ varying from 1.1 V to 2.0 V, *t*_*d*_ is calculated as defined above. *t*_*d*_ is found to decrease exponentially with an increase of *V*_*A*_ as shown in Fig. 4a. This voltage-dependent delay time characteristic can be expressed as:

1 $${t}_{d}={c}_{1}\times \exp \{-(\frac{{V}_{A}-{V}_{T}}{{V}_{T}})\times (\frac{{c}_{2}}{{V}_{T}})\}$$

where, c_1_ and c_2_ are constants^30^, and the values of c_1_, c_2_ for the best fit of our data are 2239 and 8.8 respectively. It can be observed from Fig. 4a that for voltage pulses of 1.1 V – 1.3 V, *t*_*d*_ is of the order of hundreds of µs. However, for voltage pulses of 1.6 V and above, *t*_*d*_ reduces significantly down to the ns-timescale and further to the ps-timescale as plotted on a log scale in inset of Fig. 4a. Upon applying a sufficiently high voltage (2.0 V), *t*_*d*_ reduces down to a value smaller than 50 ps, which is the measurement limit of the experimental setup. To obtain further insight on ultrafast threshold switching approaching the measurement limit of the test system (*t*_*d*_ < 50 ps), the applied voltage pulse (*V*_*A*_ of 2 V, rise time of 1 ns, fall time of 1 ns and pulse-width of 5 ns) and the corresponding device response is shown in Fig. 4b. It can be clearly observed that upon application of voltage pulse of 2.0 V (i.e. twice of *V*_*T*_), the device switches instantaneously at the applied voltage of 1 ± 0.1 V (equivalent to *V*_*T*_ of the device). Thus, within the measurement limit of the existing test system, we observed an almost instantaneous switching of the IST device. The ultimate switching speed of IST device may thus be even faster, as the experimental findings are limited by the test system capabilities.

The *E*_*T*_ of PCM device plays an important role in governing the switching transition from amorphous off to conducting state. The *E*_*T*_ of 25 V/µm for IST devices is lower than that of conventional Ge_2_Sb_2_Te_5_ and GeTe devices^9,23^. Most of the phase change materials including various GeTe-Sb_2_Te_3_ tie-line compositions possess a trade-off between the switching speed and stability of the amorphous phase. Achieving the combination of better thermal stability and fast switching speed in PCM devices has been challenging. The present study illustrates that IST could be a viable alternative. Its excellent property portfolio of better thermal stability, large resistance contrast, single phase crystallization, and ultrafast threshold switching make IST a promising candidate for realizing high-speed non-volatile PCM devices.

Understanding of both kinetic and material properties is essential for the development of memory devices with universal characteristics. Our results demonstrate better thermal stability and large resistivity contrast between the amorphous and crystalline phases of IST. In addition to this, the rapid single phase crystallization enables faster programming of IST devices. Also, the threshold voltage (or electric field) required for switching IST device is found to be lower as compared to other phase change materials. Moreover, the voltage-dependent threshold switching dynamics of IST devices using time-resolved current-voltage measurement demonstrate an exponential reduction in *t*_*d*_ upon increasing the *V*_*A*_. An ultrafast threshold switching with *t*_*d*_ less than 50 ps has been achieved upon applying a voltage pulse of twice of *V*_*T*_. This peculiar combination of ultrafast threshold switching and better thermal stability of IST devices paves a way for enabling PCM devices with extraordinary performance characteristics.

## Methods

### Device fabrication

For material characterizations, IST thin films were sputter deposited from a stoichiometric sputtering target purchased from *ACI Alloys Inc., USA* on pre-cleaned Si and SiO_2_ substrates using DC/RF magnetron sputtering. For investigation of threshold switching dynamics, PCM devices were fabricated in a sandwich type structure i.e. a thin IST layer is placed between top and bottom electrodes (T.E. and B.E. respectively) on a pre-cleaned SiO_2_ substrate. Initially, a 50 nm thin W layer is sputter deposited as B.E. at a constant DC power of 60 W with a deposition rate of 0.066 nm/s. Further, 40 nm thin active layer of IST was sputter deposited at a constant DC power of 24 W with a deposition rate of 0.098 nm/s. The sputtering chamber was evacuated down to 2 × 10^−6^ mbar before deposition. The Ar flow and process pressure during the deposition were 20 sccm and 6.2 × 10^−3^ mbar respectively and the source to substrate distance was 450 mm. Lastly, T.E. of 5 nm Cr followed by 100 nm Au was deposited using thermal evaporation technique with deposition rates of 0.05 nm/s and 0.2 nm/s respectively. Cr was deposited to avoid the diffusion of Au into the active layer.

### Temperature-dependent resistivity measurement

Thin film samples were placed inside a tubular furnace for annealing and measurements were carried out in four-point probe configuration using Van-der-Pauw method. A steady Ar flow was maintained during the entire process. To measure the resistance of the film, a source and measure unit (SMU, *Keithley*) was used to source the current and measure the voltage in different configurations of Van-der-Pauw method.

### Time-resolved current voltage measurement

To study the threshold switching dynamics in fast timescale and capture the device response on the ps timescale, a programmable electrical test (PET) system is utilized. This setup essentially consists of (i) Arbitrary Waveform Generator (AWG, *Agilent Technologies —81160* *A*) that can generate voltage pulses up to 5 V with minimum rise/fall time of 1 ns and pulse-width as short as 1.5 ns, (ii) Digital Storage Oscilloscope (DSO, *Teledyne Lecroy–Wavepro 735zi-A*) having a bandwidth of 3.5 GHz and data resolution of 50 ps at a sampling rate of 20 GSa/s, and (iii) Custom-designed probe-station with high frequency contact boards enabling measurement of ultrafast transient characteristics with a resolution of 50 ps.

## Supplementary information

Exploring ultrafast threshold switching in In3SbTe2 phase change memory devices
