# Supplementary material for: Exploring ultrafast threshold switching in In3SbTe2 phase change memory devices
Source: Sci Rep. 2019 Dec 17;9:19251. doi: 10.1038/s41598-019-55874-5 (PMC6917803; doi:10.1038/s41598-019-55874-5)
Supplement: Supplementary file 1 — Exploring ultrafast threshold switching in In3SbTe2 phase change memory devices [file 41598_2019_55874_MOESM1_ESM.docx]

Supplementary Information

Exploring ultrafast threshold switching in In_3_SbTe_2_ phase change memory devices

**Nishant Saxena, Christoph Persch, Matthias Wuttig and Anbarasu Manivannan**

Correspondence should be addressed to A.M. (E-mail: anbarasu@iitm.ac.in).

**Supplementary Information:**

Supplementary Notes (1-4)

Supplementary Figures (1-9)

**SUPPLEMENTARY NOTE 1: Thermal stability of amorphous phase**

Improved thermal stability of IST as compare to sevaral other phase change materials has been validated using temperature dependent resistivity measurement and x-ray measurement techniques as described below.

**Temperature dependent resistivity measurement**

Thin IST films of various thicknesses have been annealed upto 325 °C and corresponding change in their resistivity is measured as shown in Fig. S1. It clearly demonstrates a pronounced dependence of phase transition/crystallization temperature with the variation of film thicknesses being a stable resistivity in the amorphous phase. Therefore, it is worth mentioning here that the increase of crystallization temperature (T_c_) for low thicknesses reveals that IST has prominent scaling properties. Further, the increase in crystallization temperature for lower film thickness could be due to increase in surface-area-to-volume ratio of the film leading to increased interface effects during amorphous to crystalline transformation^31^.


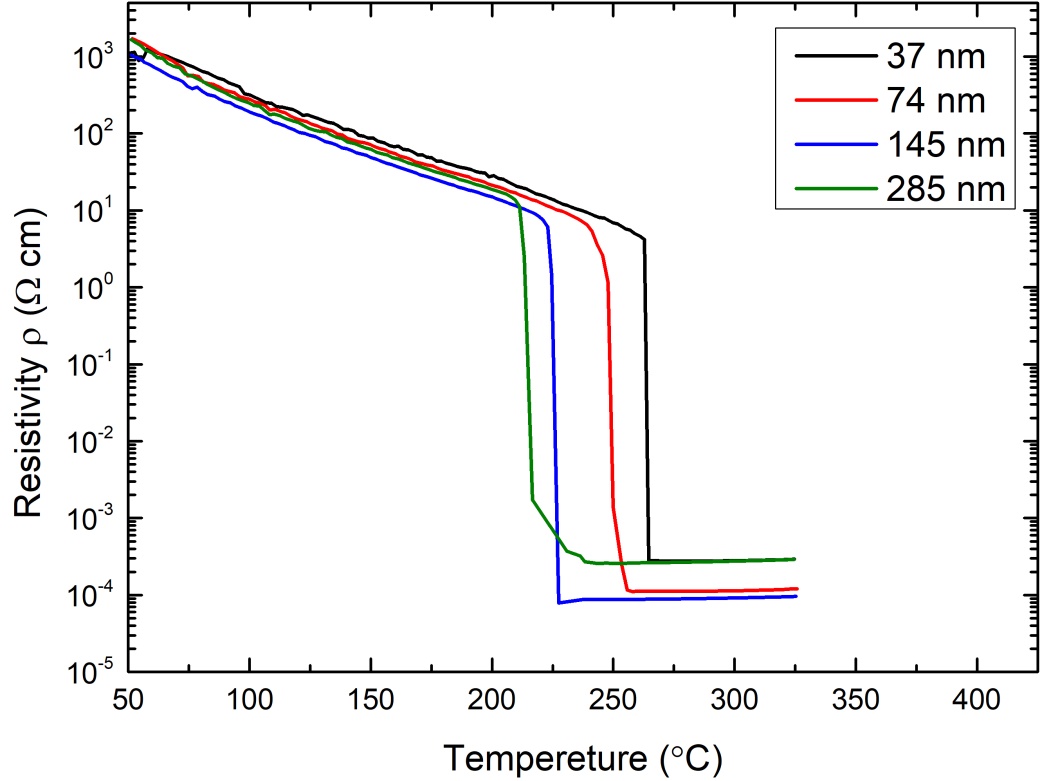


**Supplementary Figure S1.** **Thickness dependence on crystallization temperature:** Temperature dependent resistivity measurement of IST thin films demonstrating a thickness dependence on crystallization temperature.

**Activation Energy (E_a_)**

Activation energy of conduction is calculated from temperature dependent resistivity plot as shown in Fig. S2. IST shows higher activation energy than conventional Ge_2_Sb_2_Te_5_ (0.38 eV) and AgInSbTe (0.31 eV) materials^32^. Thus, higher activation energy also demonstrate better thermal stability of IST than other phase change materials.


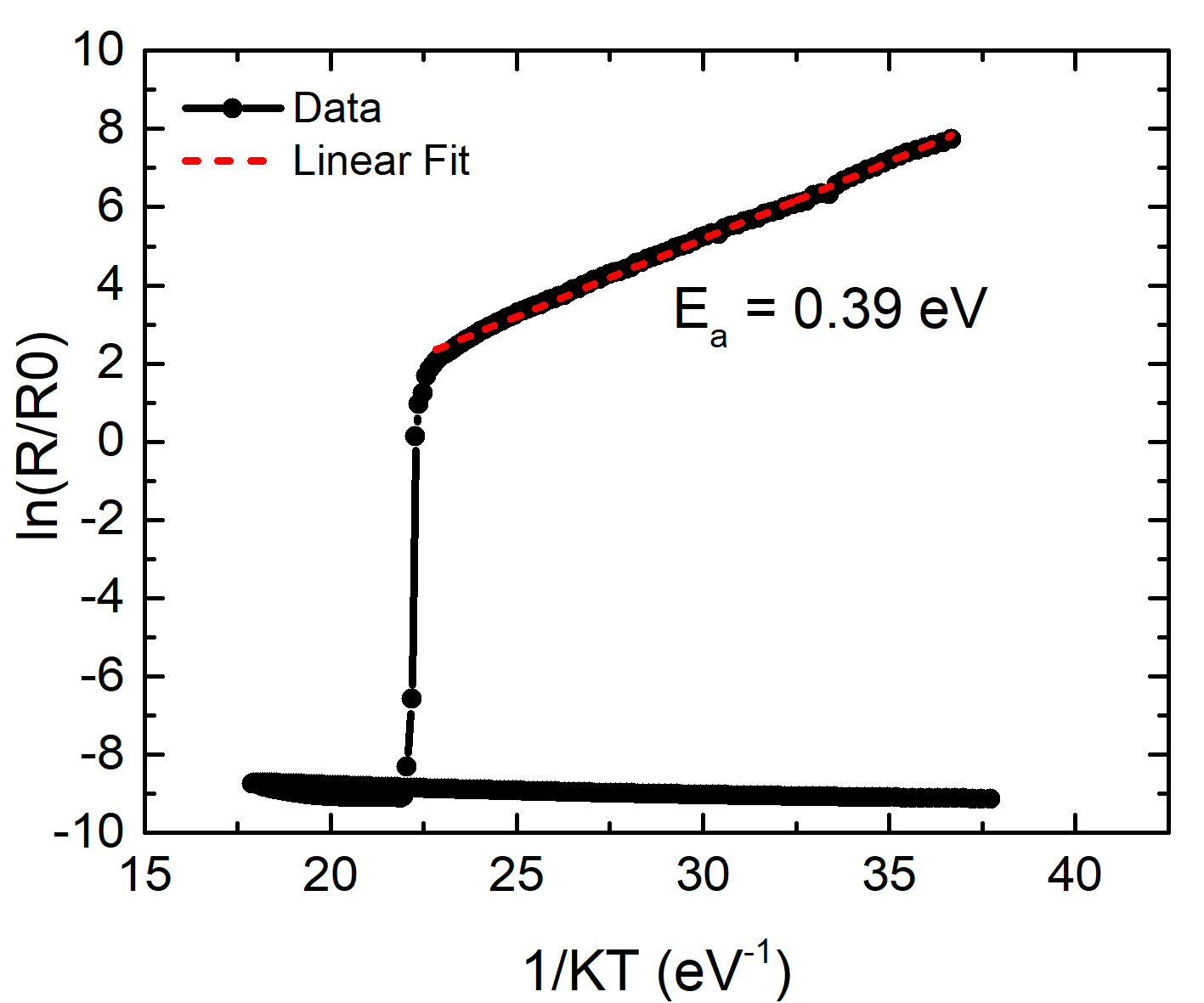


**Supplementary Figure S2. Activation energy of IST:** Activation energy of IST (0.39 eV) is higher than conventional AgInSbTe and Ge_2_Sb_2_Te_5_ materials indicating better thermal stability of IST.

**X-ray Reflectivity Measurement**

Figure S3 shows the XRR spectra of the as-deposited IST film and thermally annealed at 275 ºC for 15 min. From a comparison of the spectra, a clear shift in the critical angle for total external reflection becomes visible. Such a shift towards higher angles indicates an increase in density, since the critical angle for total external reflection is proportional to the square root of density.


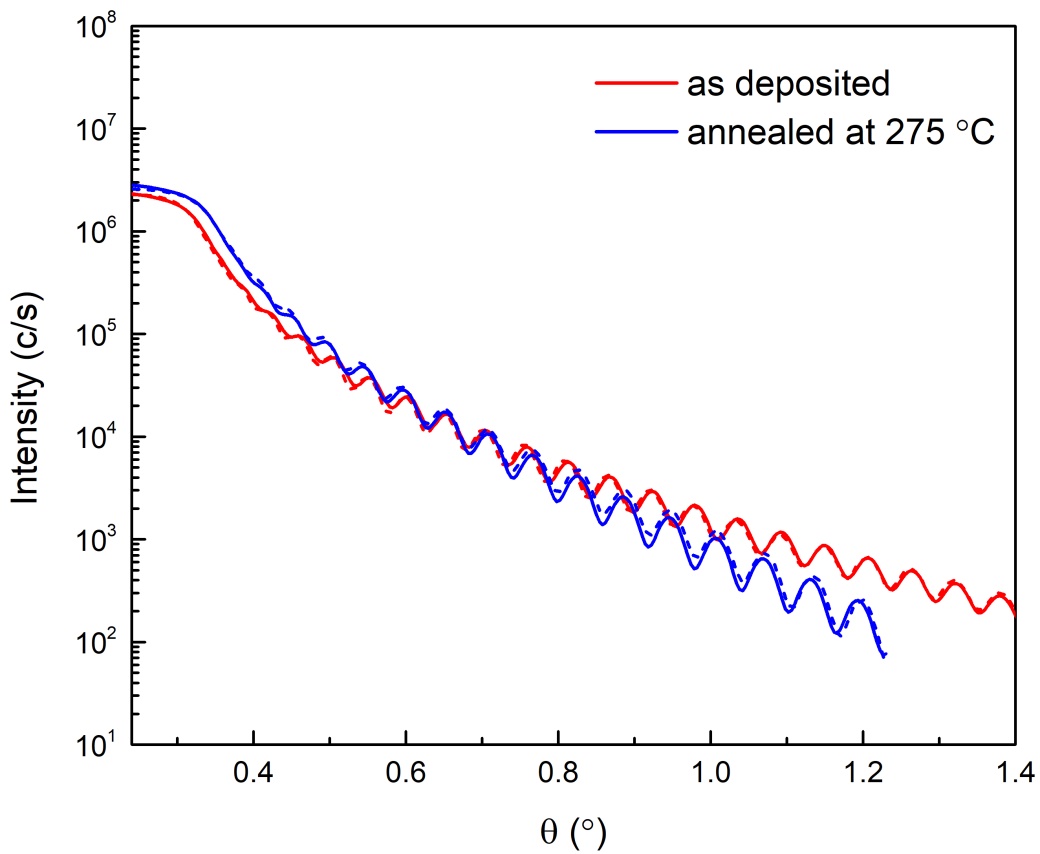


**Supplementary Figure S3.** **X-ray Reflectivity Measurement:** XRR scans of thin IST films as deposited (red colored line) and thermally annealed at 275 ºC (blue colored line). The dashed lines indicate the simulated curves.

Further, Fig. S4 depicts the normalized variations in the film thickness and density at different annealing temperatures. Interestingly, it is observed that the film thickness and density remains fairly constant in the as-deposited amorphous phase (thickness is 74 ± 0.15 nm, density is 6.17 ± 0.01 gm.cm^-3^) up to the crystallization temperature. Above 220 ºC, the film thickness and density change to 67.5 ± 0.2 nm and 6.75 ± 0.02 g.cm^-3^ that corresponds to a crystalline In_3_SbTe_2_ phase and remains invariant until 325 ºC (beyond this, material evaporation takes place and hence XRR measurement becomes difficult) suggests a stable crystalline structure. The observed change in film thickness and density between the amorphous and crystalline phase is approximately 8.5 %. The increase in density implies a contraction in volume, which corresponds to a reduction in the film thickness. To determine whether there is any material loss during annealing, the product of normalized density and thickness change was calculated for each annealing temperature. The value of mass conservation is approximately equal to 1.0 with a deviation less than 1 %.


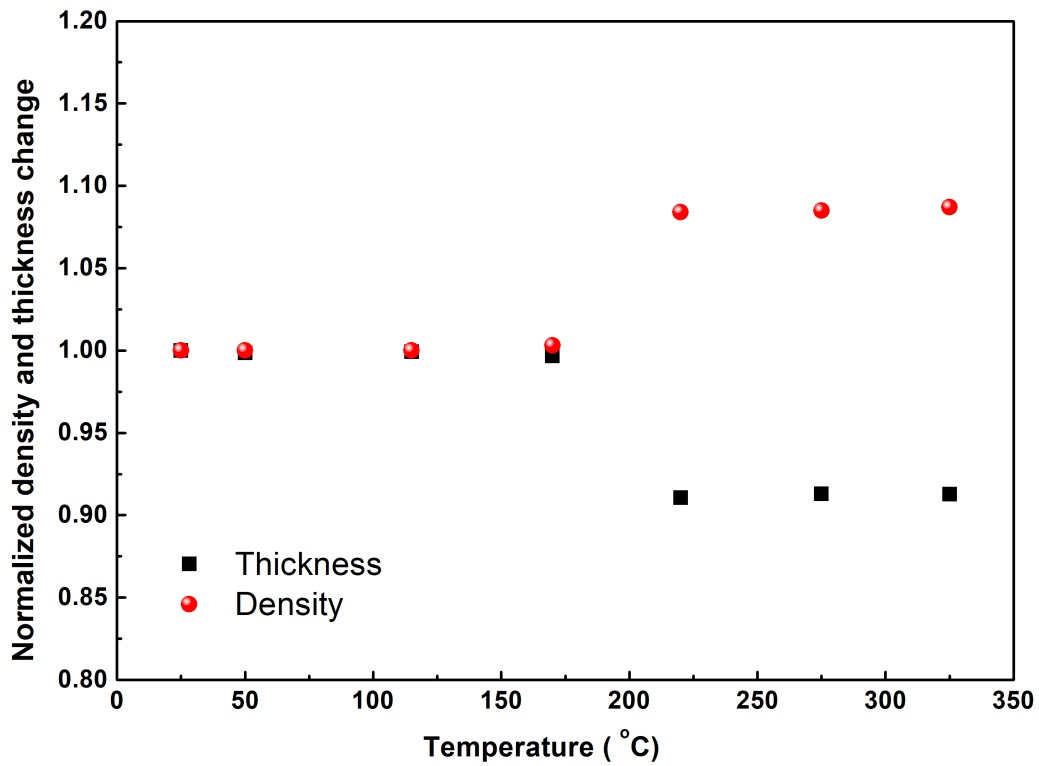


**Supplementary Figure S4.** **Normalized density and thickness change:** Density and thickness of material on various annealing temperatures.

**SUPPLEMENTARY NOTE 2: Identifying threshold voltage through time-resolved Current-Voltage measurement**

In order to identify *V_T_* of the device, a triangular voltage pulse, having an amplitude of 1.2 V and long leading, trailing edges of 10 µs is applied to the device as shown in Fig. S5. Threshold voltage is found to be 1 ± 0.1 V. The *V_T_* is measured for various devices and pulse-widths as shown below:


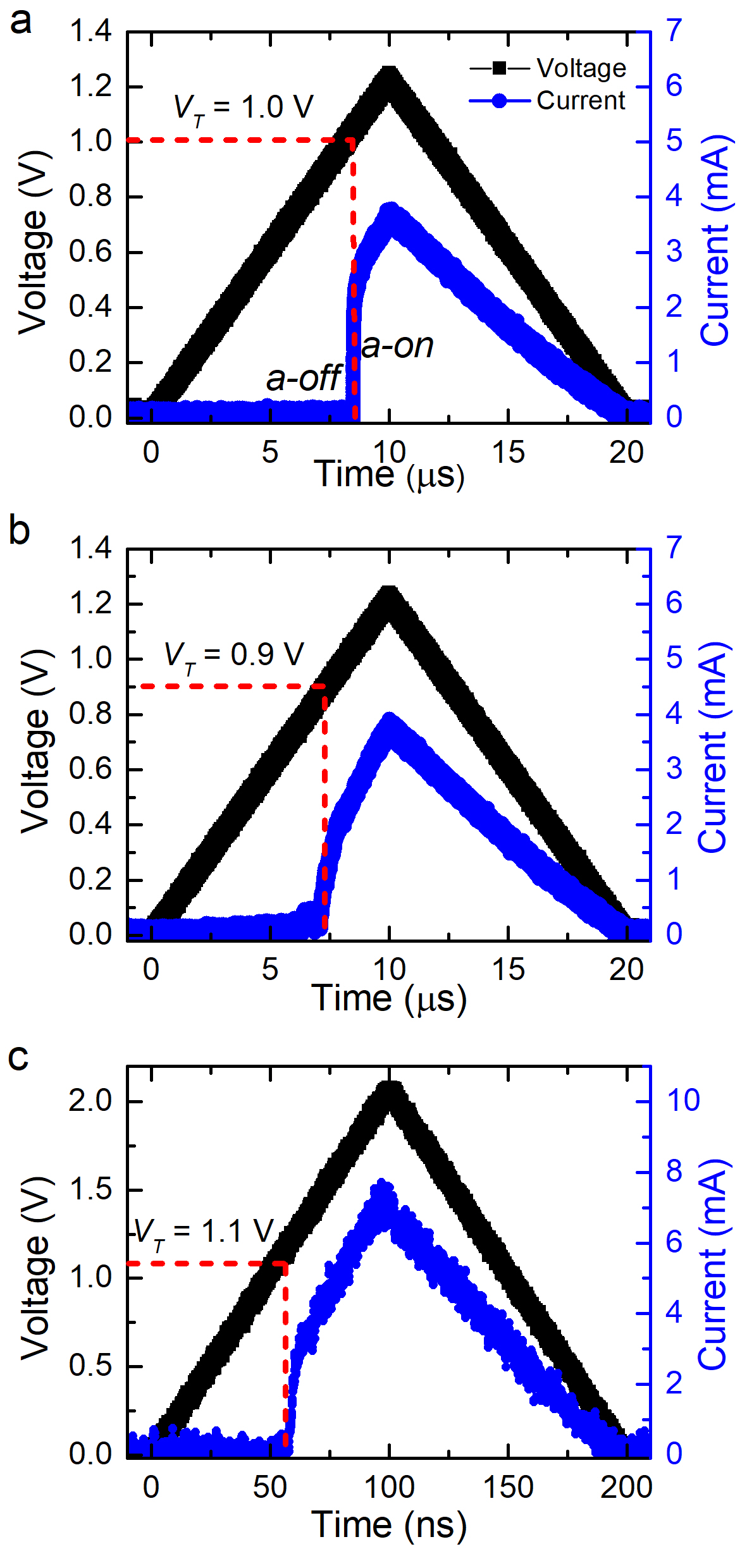


**Supplementary Figure S5. Measurement of threshold voltage:** Time-resolved measurement of current-voltage shows the threshold voltage 1 ± 0.1 V for different devices and pulses.

Furthermore, Fig. S6 shows current-voltage characteristics of IST device. The device current (in log scale) shows more than three orders of magnitude change beween as-deposited amorphous and crystalline (set) states.


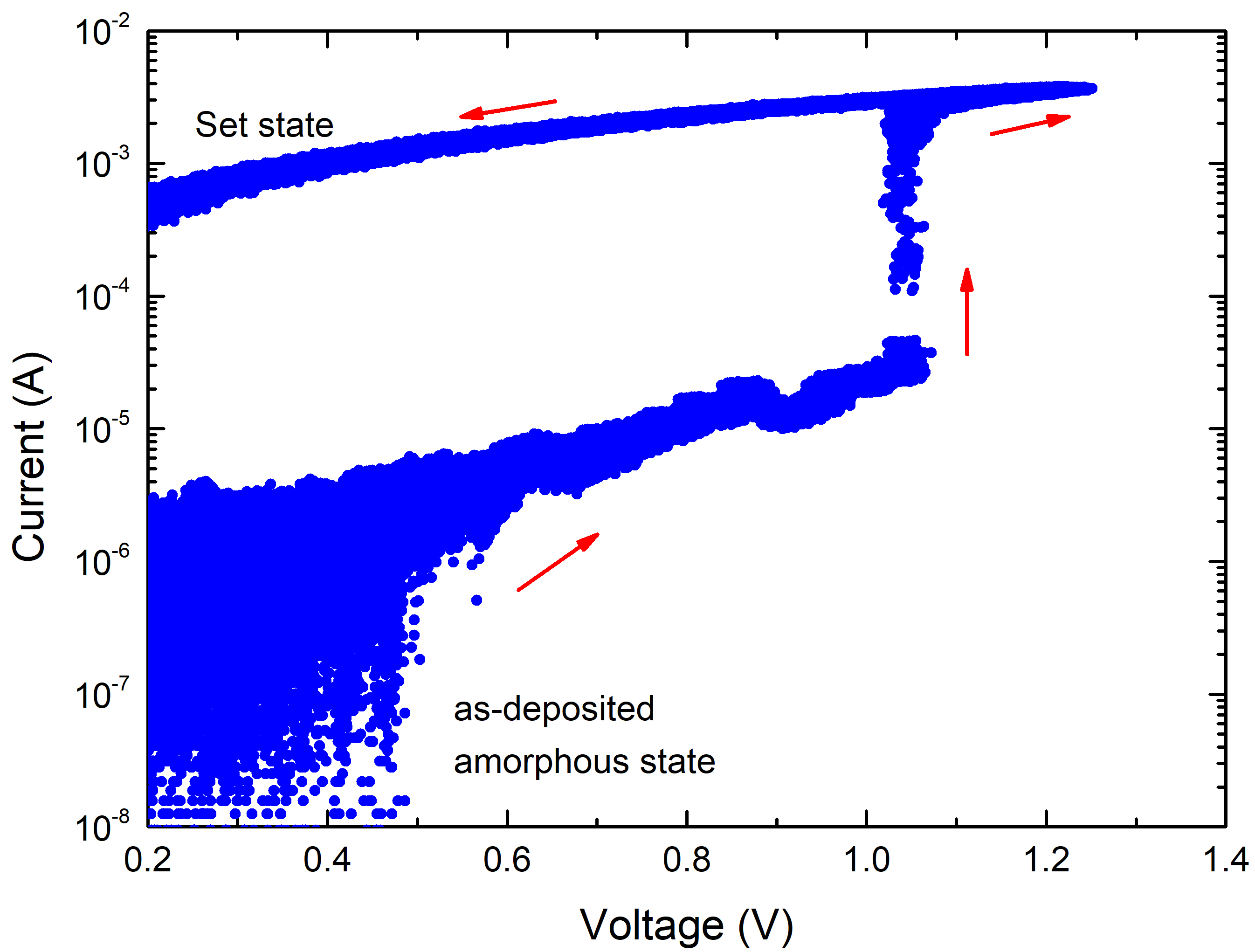


**Supplementary Figure S6. Current-voltage characteristics of IST device.** I-V characteristics demonstrates more than three orders of magnitude change beween as-deposited amorphous and crystalline (set) states.

**SUPPLEMENTARY NOTE 3: Measurement of delay time and device current during delay time**

Delay time is measured as the time duration between the device experiencing *V_T_* and a sharp rise in *I_d_*. Precise measurement of delay time is shown for two different cases in Fig. S7: i) case A, the V_A_ is merely equal to V_T_ and threshold switching takes place in plateau region, ii) case B when V_A_ >> V_T_ and the device exhibits threshold switching during the leading edge (rise time) of the applied voltage pulse. Such precise measurement is essential, when the threshold switching takes place during the leading edge (rise time) of V_A_. Therefore, in order to measure the delay time precisely, it is important to identify the time between the starting point at which the device experiences *V_T_* and up to initiation of steep rise in the *I_d_*.

**
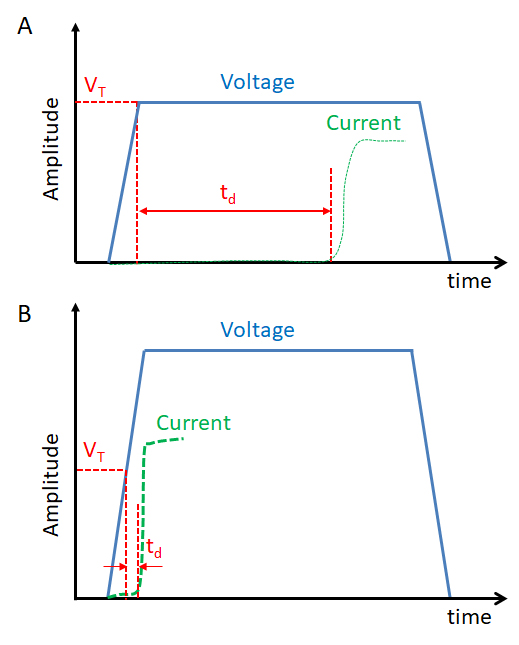
**

**Supplementary Figure S7. Measurement of delay time:** (A) V_A_ = V_T_, device switches in plateau region (B) V_A_ >> V_T_, device switches within leading edge (rise time).

**Device current during delay time**

Physical mechanism of threshold switching has been subjected to debates in literature. Some researchers believed it to be a thermal process, whereas most of them believe it to be an electronic phenomenon. Our experimental results show that device current remains fairly constant over a long time when a sharp (rise time, fall time of 1 ns) voltage pulse of amplitude 1.1 V and pulse width of 1 ms is applied to the device as shown in Fig. S8. This indicates that the off state current does not contribute to any significant increase in temperature and therefore, strongly supports the electronic nature of threshold switching.


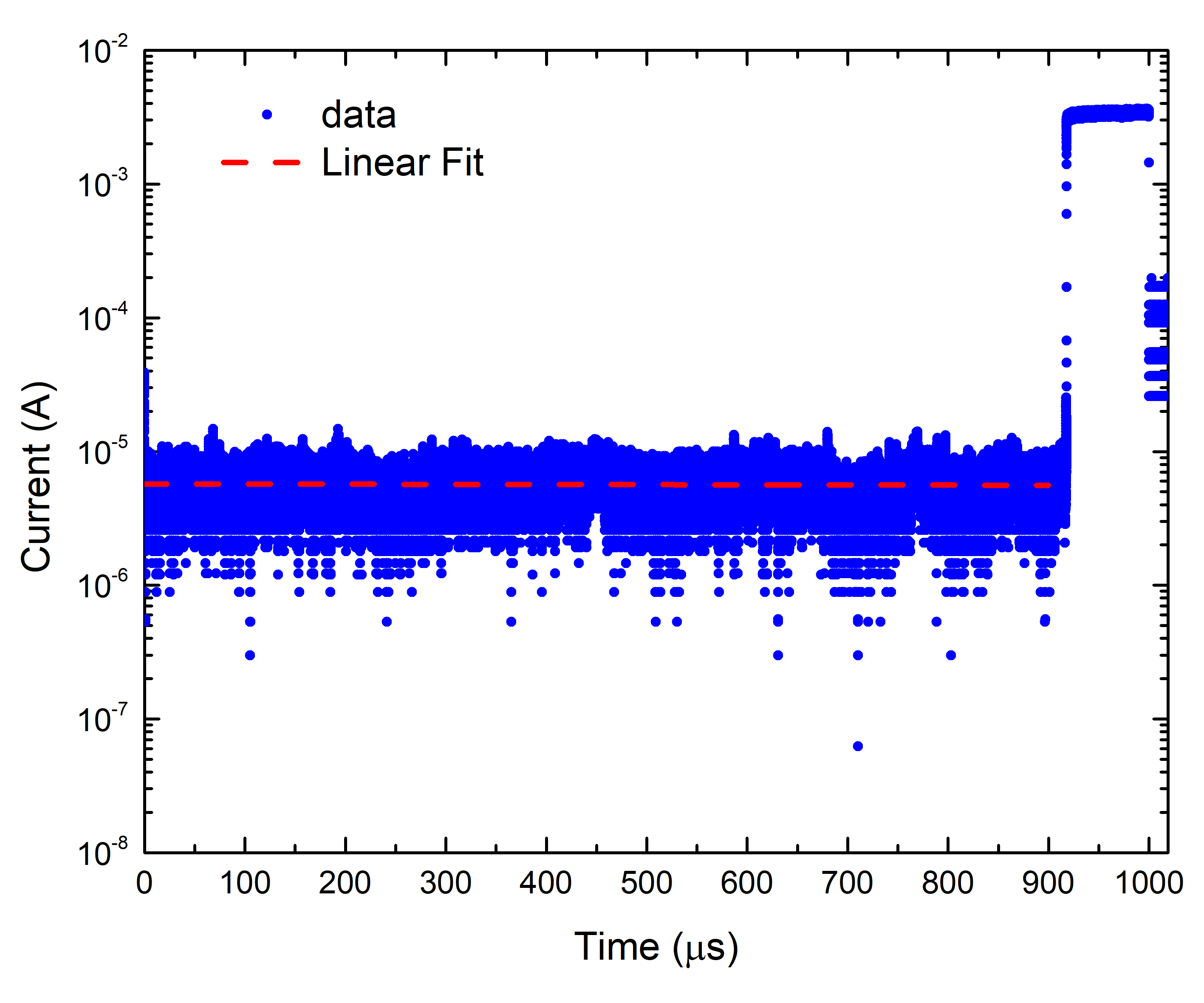


**Supplementary Figure S8.** **Constant current during delay time:** Device current remains constant during the delay time even after realizing the threshold voltage for more than 900 µs.

**SUPPLEMENTARY NOTE 4: Ultrafast threshold switching with delay time ≤ 50 ps**

Initially, device is in as-deposited amorphous state as validated by a read pulse measuring device resistance ~ 10 MΩ. Upon applying a voltage pulse of 2 V (i.e. twice of V_T_), device switches extreamly fast with a delay time of less than 50 ps (limited to the time-resolution of experimental setup). Device resistance drops down to ~ 500 Ω indicating the set state, which is confirmed by subsequent read pulse.


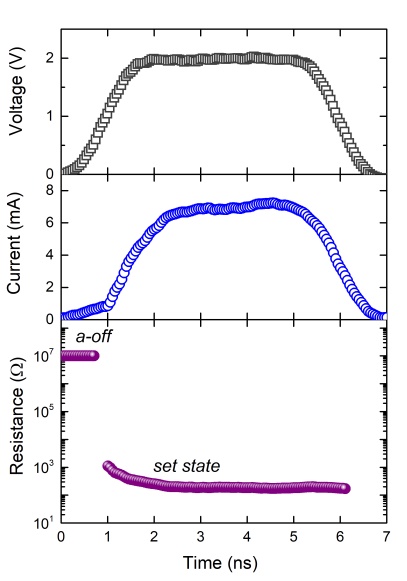


**Supplementary Figure S9. Ultrafast threshold switching:** Threshold switching with a delay time < 50 ps and set operation demonstrating more than 4 orders of magnitude change in electrical resistance of the device.

**References**

1. Zhang, W., Mazzarello, R., Wuttig, M. & Ma, E. Designing crystallization in phase-change materials for universal memory and neuro-inspired computing. *Nat. Rev. Mater.* **4,** 150-168 (2019).
2. Li, X. B., Chen, N. K.,Wang, X. P. & Sun, H. B. Phase-change superlattice materials toward low power consumption and high density data storage: microscopic picture, working principles, and optimization. *Adv. Func. Mater.* **28,** 1803380 (2018).
3. Wuttig, M. & Yamada, N. Phase-change materials for rewriteable data storage. *Nat. Mater.* **6,** 824-832 (2007).
4. Pandey, S. K. & Manivannan, A. Extremely high contrast multi-level resistance states of In_3_SbTe_2_ device for high density non-volatile memory applications. *Physica Status Solidi: Rapid Research letter* **11,** 1700227 (2017).
5. Kau. D. et al. A stackable cross point phase change memory. *Tech. Dig. - Int. Electron Devices Meet. IEDM* (2009). DOI: 10.1109/IEDM.2009.5424263
6. Lai, S. & Lowrey, T. OUM - A 180 nm nonvolatile memory cell element technology for stand alone and embedded applications. *Tech. Dig.- Int. Electron Devices Meet. IEDM* (2001). DOI: 10.1109/IEDM.2001.979636
7. Wang, Y. et al. High thermal stability and fast speed phase change memory by optimizing GeSbTe with Scandium doping. *Scripta Materialia* **164**, 25-29 (2019).
8. Wang, J. et al. Unconventional two-dimensional germanium dichalcogenides *Nanoscale* **10,** 7363–7368 (2018).
9. Lankhorst, M. H. R., Ketelaars, B. W. S. M. M. & Wolters, R. A. M. Low-cost and nanoscale non-volatile memory concept for future silicon chips. *Nat. Mater.* **4,** 347–352 (2005).
10. Kim, Y. T. & Kim, S. Il. Comparison of thermal stabilities between Ge-Sb-Te and In-Sb-Te phase change materials. *Appl. Phys. Lett.* **103,** 121906 (2013).
11. Maeda, Y., Andoh, H., Ikuta, I. & Minemura, H. Reversible phase-change optical data storage in InSbTe alloy films. *J. Appl. Phys.* **64,** 1715 (1988).
12. Deringer, V. L. et al. A chemical link between Ge–Sb–Te and In–Sb–Te phase-change materials. *J. Mater. Chem. C* **3,** 9519–9523 (2015).
13. Ahn, J.-K., Park, K.-W., Jung, H.-J. & Yoon, S.-G. Phase-change InSbTe nanowires grown in situ at low temperature by metal-organic chemical vapor deposition. *Nano Lett.* **10,** 472–477 (2010).
14. Kim, Y. I., Kim, E. T., Lee, J. Y. & Kim, Y. T. Microstructures corresponding to multilevel resistances of In_3_Sb_1_Te_2_ phase-change memory. *Appl. Phys. Lett.* **98,** 091915 (2011).
15. Rao, F. et al. Reducing the stochasticity of crystal nucleation to enable subnanosecond memory writing. *Science* **358,** 1423–1427 (2017).
16. Shukla, K., Saxena, N., Durai, S. & Manivannan, A. Redefining the speed limit of phase change memory revealed by time-resolved steep threshold- switching dynamics of AgInSbTe devices. *Sci. Rep.* **6**, 37868 (2016).
17. Loke, D. et al. Breaking the speed limits of phase-change memory. *Science* **336,** 1566–1569 (2012).
18. Ovshinsky, S. R. Reversible electrical switching phenomena in disordered structures. *Phys. Rev. Lett.* **21,** 1450–1453 (1968).
19. Adler, D., Shur, M. S., Silver, M. & Ovshinsky, S. R. Threshold switching in chalcogenide glass thin films. *J. Appl. Phys.* **51,** 3289 (1980).
20. Ielmini, D. Threshold switching mechanism by high-field energy gain in the hopping transport of chalcogenide glasses. *Phys. Rev. B* **78,** 035308 (2008).
21. Buckley, W. D. & Holmberg, S. H. Evidence for critical-field switching in amorphous semiconductor materials. *Phys. Rev. Lett.* **32,** 1429–1432 (1974).
22. Zalden, P. et al. Picosecond electric-field-induced threshold switching in phase-change materials. *Phys. Rev. Lett.* **117,** 067601 (2016).
23. Bruns, G. et al. Nanosecond switching in GeTe phase change memory cells. *Appl. Phys. Lett.* **95,** 043108 (2009).
24. Wang, W. J. et al. Fast phase transitions induced by picosecond electrical pulses on phase change memory cells. *Appl. Phys. Lett.* **93,** 043121 (2008).
25. Anbarasu, M., Wimmer, M., Bruns, G., Salinga, M. & Wuttig, M. Nanosecond threshold switching of GeTe_6_ cells and their potential as selector devices. *Appl. Phys. Lett.* **100,** 143505 (2012).
26. Kang, D. H. et al. Time-resolved analysis of the set process in an electrical phase-change memory device. *Appl. Phys. Lett.* **87,** 253505 (2005).
27. Wimmer, M. & Salinga, M. The gradual nature of threshold switching. *New J. Phys.* **16,** 113044 (2014).
28. Pandey, S. K. & Manivannan, A. A fully automated temperature-dependent resistance measurement setup using van der Pauw method. *Rev. Sci. Instrum.* **89,** 033906 (2018).
29. Shukla, K. D., Saxena, N., & Manivannan, A. An ultrafast programmable electrical tester for enabling time-resolved, sub-nanosecond switching dynamics and programming of nanoscale memory devices. *Rev. Sci. Instrum.* **88,** 123906, (2017).
30. Schöll E. *Nonequilibrium Phase Transitions in Semiconductors: Self-organization Induced by Generation and Recombination Processes*. (Springer-Verlag Berlin Heidelberg, 1987).
31. Chopra, K. L. & Bahl, S. K. Amorphous versus crystalline GeTe films. I. Growth and structural behavior. *J. Appl. Phys.* **40**, 4171 (1969).
32. K. Shportko et al. Resonant bonding in crystalline phase-change materials. *Nat. Mater.* **7**, 653 (2008).
